# Impact of adding additional providers to resident workload and the resident experience on a medical consultation rotation

**DOI:** 10.1186/s12909-017-0874-7

**Published:** 2017-02-22

**Authors:** Michele Fang, Eric Linson, Manish Suneja, Ethan F. Kuperman

**Affiliations:** 1 Department of Internal Medicine, Carver College of Medicine, University of Iowa, Iowa City, IA USA; 2 Department of Internal Medicine, Perelman School of Medicine, University of Pennsylvania, Philadelphia, PA USA; 3 Section of Hospital Medicine, Hospital of the University of Pennsylvania, Department of Medicine, Section of Hospital Medicine, 3400 Spruce Street, Maloney Building, 5th floor, Suite 5033, Philadelphia, PA 19104 USA

**Keywords:** Workload, Internship and residency, Internal medicine, Preoperative care, Graduate medical education, Nurse practitioners

## Abstract

**Background:**

Excellence in Graduate Medical Education requires the right clinical environment with an appropriate workload where residents have enough patients to gain proficiency in medicine with optimal time for reflection. The Accreditation Council for Graduate Medical Education (ACGME) has focused more on work hours rather than workload; however, high resident workload has been associated with lower resident participation in education and fatigue-related errors. Recognizing the potential risks associated with high resident workload and being mindful of the costs of reducing resident workload, we sought to reduce residents’ workload by adding an advanced practice provider (APP) to the surgical comanagement service (SCM) and study its effect on resident satisfaction and perceived educational value of the rotation.

**Methods:**

In Fiscal Year (FY) 2014 and 2015, an additional faculty member was added to the SCM rotation. In FY 2014, the faculty member was a staff physician, and in FY 2015, the faculty member was an APP.. Resident workload was assessed using billing data. We measured residents’ perceptions of the rotation using an anonymous electronic survey tool. We compared FY2014-2015 data to the baseline FY2013.

**Results:**

The number of patients seen per resident per day decreased from 8.0(SD 3.3) in FY2013 to 5.0(SD 1.9) in FY2014 (*p* < 0.001) and 5.7(SD 2.0) in FY2015 (*p* < 0.001). A higher proportion of residents reported “just right” patient volume (64.4%, 91.7%, 96.7% in FY2013, 2014, 2015 respectively *p* < 0.001), meeting curricular goals (79.9%, 95.0%, 97.2%, in FY2013, 2014 and 2015 respectively *p* < 0.001), and overall educational value of the rotation (40.0%, 72.2%, 72.6% in FY2013, 2014, 2015 respectively, *p* < 0.001).

**Conclusions:**

Decreasing resident workload through adding clinical faculty (both staff physician and APPs) was associated with improvements on resident perceived educational value and clinical experience of a medical consultation rotation.

**Electronic supplementary material:**

The online version of this article (doi:10.1186/s12909-017-0874-7) contains supplementary material, which is available to authorized users.

## Background

The majority of Accreditation Council for Graduate Medical Education (ACGME) efforts to enhance patient safety and decrease resident fatigue have been focused on reducing residents’ duty hours [1]. One consequence to decreasing duty hours is “work compression,” the expectation that residents complete a fixed amount of work within fewer hours. Work compression increases perceived resident workload, prolonged occupational stress, and burnout with high job demands and low individual autonomy. High resident workload has been associated with decreased participation in educational activities,[2] increased fatigue-related medical errors,[3] and higher patient mortality [4].

Inpatient volume (census) for individual residents and the resident team is a major component of residents’ workload [5]. Biaggi et al. found that one third of the medicine residents felt overburdened by the workload often or most of the time and 69% rated their work intensity as “high” (“too high” in 3%) [5]. One study that developed an Integrated Teaching Unit (ITU) with reduced clinical load was associated with improvements in resident satisfaction and more time for learning; however, there was no improvement in length of stay (LOS) or readmissions and there was associated increased costs for hiring additional staff [6].

Two studies increased the number of residents on the general medicine service. These two studies had no improvements in subject exam scores or direct contact with patients though there was in perceived resident satisfaction of the overall quality of the clerkship, improvement in rounding with attendings, LOS, ICU days, and quality of discharge summaries [7, 8]. In contrast, cost-neutral programs such as census caps and geographical rounding did not decrease the mean midnight census and had no effect on patient safety outcomes [9].

As of 2006, clinically active APPs comprise one sixth of the US medical workforce with approximately 11,000 new APP graduates each year [10]. Prior studies have found that academic medical centers increased use of APPs because of ACGME resident duty hour restrictions, to increase patient throughput, increase patient access, and improve continuity of care [11]. A systematic review of APP outcomes found that APP provide care that has equivalent rates of patient satisfaction, self-reported perceived health, functional status, glucose control, blood pressure, emergency department visits, hospitalization and mortality, and better serum lipid control [11]. However, other outcomes such as resident education and inpatient quality metrics have not been well-studied.

Recognizing the potential risks associated with high resident workload and being mindful of the costs of reducing resident workload, we sought to reduce residents’ workload by adding an APP to the surgical comanagement service. The aim of this study was to examine the effect of this intervention on residents’ perceptions of their workload and surgical comanagement rotation.

## Methods

### Setting and participants

The University of Iowa Hospitals and Clinics (UIHC) is a 700-bed, tertiary-care, teaching hospital located in a suburban, US community. The UIHC Internal Medicine Residency is a 3-year accredited program with 90 internal medicine, medicine preliminary, and medicine/psychiatry residents. All residents have a 4-week SCM rotation during their training. In addition, oral surgery, psychiatry, and preliminary interns also serve on SCM.

The SCM rotation provides inpatient comanagement services and traditional medical consultation services to surgical specialties (e.g. orthopedics) as well as neurology and psychiatry at the UIHC. The inpatient services cover new and follow up consults. Afternoon preoperative risk assessment and optimization clinics are also scheduled Monday through Thursday and covered by the SCM teams to evaluate patients prior to both elective and time sensitive surgeries.

**Fig. 1:**
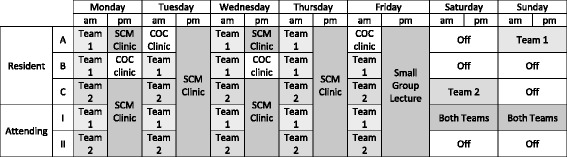
Sample staffing assignments. The table illustrates a sample 1-week calendar of resident and attending assignments on the SCM rotation. In this example, there are 3 residents and 2 attending physicians assigned. Residents A and B are assigned to Team 1, Resident C is assigned to Team 2. Residents A and B are categorical, and have 2 half-days of COC clinic. Resident C is a preliminary intern who does not have COC clinic. Weekend days rotate between residents. At the midpoint of a 4-week rotation, residents would switch SCM teams. Attending physicians served in 2-week rotations on a single SCM service

There are two inpatient SCM services with two attending physicians. Between one and five internal medicine residents, off-service, and preliminary interns rotate on the service during each block. The internal medicine chief residents and scheduling assistants allocate residents to the services. All residents average 1 day off per week. Categorical residents also have 2 half-days of continuity of care clinics during their rotation. One resident covers each weekend day (2 workdays). Please see Fig. 1 for a sample staffing schedule. Staff physicians can see a portion of the patients without resident involvement, but must see and examine all resident patients.

### Rotation description

The baseline included surveys returned 1 year prior to the intervention (FY2013, 7/2012-6/2013). The intervention study included 2 years during the intervention (FY2014-FY2015, 7/2013-6/2015). From 7/1/2013-2/28/2014, an additional faculty member was added to staff the preoperative clinic. From 3/1/2014 to 6/30/2015, the additional support in the preoperative clinic was staffed by an APP. The APP chose not to supervise residents.

### Rotation evaluation

We used billing data based on billing charges to determine resident and staff workload. We collected data from an internally developed data warehouse. (HEDI, Iowa City, IA) See Additional file 1 for billing codes applicable for charges. We calculated the average number of patients seen by residents per week, correcting for resident days off or in their primary-care continuity clinics. We divided the total number of bills generated by all of the residents by the number of resident workdays per week for the inpatient and outpatient service lines.

Faculty members are evaluated by trainees in their clinical rotations using an online survey (MedHub, LLC, Dexter, MI). The residents’ perception of the SCM clinical rotation was collected at the end of each residency ward rotation in aggregate so that all responses were anonymous. See Additional file 2 for the survey instrument. Residents rated the appropriateness of their workload by rating “Adequacy of patient volume,” and “Appropriateness of patient case mix.” The survey also solicited ratings for the “Appropriate balance between responsibility and supervision.” We based resident perception of the educational value of the rotation on the following items: (1) “The rotation specific curricular goals were met” and (2) “Overall educational value of this clinical activity.” Residents were also asked to identify the “strengths” and “weaknesses” of the rotation in open-ended questions. Resident free-text comments were reviewed by two investigators (EK and MF). Each comment was categorized into 4 divisions based on an a priori determined rubric of (1) educational value (eg “bread and butter medicine”, “teaching”), (2) workload (e.g. “volume”, “busy), (3) resident experience (“enjoyed”, “appreciate”), and (4) none (e.g. none, see above). Consensus was reached for each categorized comment.

Changes in residents’ workload and resident survey results were compared between the pre-intervention and post-intervention time periods using 2-tailed t-tests and chi-square analysis. We categorized survey responses by percentage of respondents strongly agreeing and agreeing for meeting rotation specific curricular goals and percentage of very good or excellent ratings for overall educational value.

A priori, we defined P values <0.05 as statistically significant. All calculations were performed using Microsoft Excel (Redmond, WA).

This research was approved by the University of Iowa IRB board and was performed in compliance with the Helsinki Declaration.

## Results

The demographics of residents rotating on SCM are described in Table 1. The vast majority of residents (92%) were categorical or preliminary internal medicine residents. Survey response rates were above 80% for all three years.

**Table 1:** Demographics of respondents

	2012–2013	2013–2014	2014–2015	All years
Assigned residents, n	43	36	60	139
Year of training				
PGY 1 n (%)	20 (47)	18 (50)	15 (25)	53 (38)
PGY 2 n (%)	16 (37)	11 (30)	23 (39)	50 (36)
PGY ≥3 n (%)	5 (12)	5 (13)	16 (26)	26 (19)
Male n (%)	28 (65)	23 (65)	44 (74)	95 (68)
Off-service n (%)	3 (7)`	3 (8)	5 (8)	11 (8)
Preliminary n (%)	2 (5)	2 (5)	1 (1)	5 (4)
Survey responses n (%)	43 (100)	33 (92)	49 (82)	125 (90)

The average number of patients seen per resident per day during the baseline year was 8.0 (SD 3.3). During the intervention, this decreased to 5.0 (SD 1.9) in FY2014 (*p* < 0.001) and 5.7 (SD2.0) in FY2015 (*p* < 0.001). Please see Table 2. There was a 21% decrease on the inpatient resident workload (*p* < 0.001) and a 53% decrease on the outpatient resident workload (*p* < 0.001) during the intervention period. Much of the decrease in resident workload in FY2015 was from the addition of senior residents in FY2015 as compared to FY2013 and FY2014. The staff saw fewer patients during FY2015 as there were more residents to see the patients in FY2013 and FY2014. In FY2014, the total number of patients seen by residents was less than the other 2 years and correspondingly, the staff saw more patients.

**Table 2:** numbers of patients seen per staff member (SD)

	2012–2013 (baseline)	2013–2014	2014–2015
Average daily resident total per resident	8.0(3.3)	5.0 (1.9)*	5.8 (2.0)*
Inpatient	6.6(2.9)	4.2 (1.7)*	4.7(1.7)*
Outpatient	1.8(0.8)	0.83 (0.51)*	1.1(0.47)*
Average daily total staff-only per staff	7.4 (5.2)	8.6 (2.2)*	5.9 (2.6)*
Inpatient	5.5 (3.9)	7.8 (2.4)*	4.0 (2.8)*
Outpatient	1.5 (0.7)	2.6 (0.7)*	1.9(1.3)*
Actual number of total resident pts	4279	2737	4352
Inpatient	3505	2389	3650
Outpatient	774	348	702
Actual number of total staff only pts	3088	5454	3050
Inpatient	887	702	1183
Outpatient	2201	3606	1867

*t test *p* <0.05 relative to 2012–2013

Resident survey responses describing their educational experiences on SCM are summarized in Table 3. The resident rating of “just right” patient volume improved from 62.8% pre-intervention to over 91% in FY2014 and 2015 (*p* < 0.001). Similarly the percentage of respondents reporting patient volume as too high decreased from 37.2% in FY2012 to <9.0% in FY2014 and 2015 (*p* < 0.001). An increasing percentage of residents also reported an appropriate case-mix (42.2% to 76.7%, *p* = 0.004). Qualitative responses (Table 4) confirmed that residents felt they were seeing more medically interesting and fewer routine patients. There were also improvements between level of responsibilities and supervision in 2013 (74.4% rated that they had always or usually had an appropriate balance between responsibility and supervision) compared to FY2014 (86.6%) (*p* = 0.08) and FY2015 (94.6%) (*p* = 0.002). In FY2013, 79.1% of the residents felt that the rotation specific goals were met. This improved to 100% in FY2014 (*p* = 0.010) and 94.5% (*p* < 0.001). In FY2013, only 37.2% felt that the overall education value of the clinical activity was either very good or excellent. This improved to 71.3% in FY2014 (*p* = 0.0010) and 72.6% in FY2015 (*p* < 0.001).

**Table 3:** Resident survey responses to clinical experience satisfaction ratings (relative to 2012–2013)

	2012–2013 (*n* = 43)	2013–2014 (*n* = 33)	2014–2015 (*n* = 49)
Adequacy of patient volume	37.2% too many	8.3% too many*	3.3% too many*
	62.8% just right	91.7% just right*	96.7% just right*
	0% too few	0% too few	0% too few
Appropriate balance between responsibility and supervision (% Always or usually)	74.4%	86.1%*	93.3%*
Appropriateness of patient case-mix (% Always or usually)	42.2%	61.1%*	76.7%*
Curricular goals were met (%agree or strongly agree)	79.9%	95.0%*	97.2%*
Overall educational value (% very good or excellent)	40.0%	72.2%*	72.6%*

*Chi Square *p* < 0.05 relative to FY 2013

**Table 4:** Resident responses as strengths and weaknesses for FY 2013–2015.

	Educational value	Workload	Resident experience	None
Strengths of rotation FY 2013 (*n* = 16)	93.8 (24.2)	25.0 (43.3)	37.5 (48.4)	0 (24.2)
Strengths of rotation FY 2014 (*n* = 22)	72.7 (42.6)	22.7 (42.6)	45.5 (49.5)	4.5 (21.2)
Strengths of rotation FY 2015 (*n* = 36)	86.1 (34.6)	13.9 (34.6)	50.0 (41.6)	0 (16.4)
Weaknesses of rotation FY2013 (*n* = 16)	55.8 (49.7)	41.2 (49.2)	55.8 (49.7)	2.9 (16.9)
Weaknesses of rotation FY 2014 (*n* = 22)	60.0 (49.0)	60.0 (49.0)	50.0 (50.0)	10.0 (30.0)
Weaknesses of rotation FY 2015 (*n* = 36)	50.0 (50.0)	27.3 (44.5)	36.4 (48.1)	18.2 (20.1)

Responses reported as % of total responses with standard deviation in parenthesis

The qualitative analysis of resident comments are summarized in Table 4. One hundred and forty-eight responses were received over the 3-year study period. Two questions were asked to the resident each year soliciting the strengths and weaknesses of the rotation. We found no significant differences between the number of responses in each category of educational value, resident experience, or workload during the control time period (FY13) and intervention time period (FY14-FY15). However, the character and tone of representative comments on workload changed after the intervention. For example, typical comments in FY2013 included statements referring to “large volume of patients” or “for once the patient volume is appropriate.” In contrast, typical FY2014 comments mention “very reasonable patient load” in FY2015. In addition, post-intervention comments highlighted the educational value of the rotation including “It is a great rotation for learning the basics of peri-operative management in both out and in patient settings” and “Great opportunity to learn more about anticoagulation, pre and post-op medication management and how to treat surgical complications.”

The APP saw more patients than the additional physician: 5.51 patients per day compared to average 4.00 patients per day (*p* < 0.001). There was no change in resident satisfaction or perceived educational value when the additional staff was an additional faculty member versus APP.

## Discussion

The additional SCM staff member decreased resident clinic workload by 2.5 patients per day. The perceived educational value improved despite no change in the core curriculum, the number of formal teaching sessions, and the physician faculty during the study period. While we encouraged residents to spend additional time reading and researching each patient, we did not quantify resident activities by time.

Past studies performed by McMahon et al. found that reduction of an intern workload on a general medicine service from 6.6 to 3.5 patients per day was associated with interns having more time for learning and higher trainee satisfaction, but it was not clear that these results would translate to a higher volume service [6]. The cost to provide this level of staffing across all academic medical centers was estimated to be $1.5 billion [12].

Although residents gain knowledge by doing, there is a continued debate between service and education. Many resident perceptions state they were seeing too many patients and spending more time with service than learning. The medical education community is still trying to balance how many patients residents need to manage to maximize their education. If too much time is spent caring for patients and documentation, residents may not feel that they are gaining knowledge from these experiences and feel that they have no time for learning and thinking about their patients [13].

Our use of an APP to decrease resident workload without additional faculty may be less costly given differences in salary and shifts worked per year. In addition, use of APP rather than faculty members may also help with physician shortages. The APP in our study also saw more patients than the faculty physicians. The effectiveness of the APP will need to be measured in terms of outcomes and cost-efficiency.

Supervision was a major emphasis of the 2011 ACGME guidelines revision, but is difficult to quantitate. Improved resident ratings for supervision were an unexpected benefit during our intervention. With additional help in the clinic, inpatient staff could more directly supervise afternoon consults.

Resident evaluations are subjective, and may not translate to objective improvements in medical knowledge and clinical performance. However, resident perceptions provide faculty direct feedback on resident understanding and priorities. Studies have shown that faculty members and residents perceptions are often not aligned [13–15]. Rose, et al. found that although both residents and faculty members agreed on the need to improve intraoperative education, there was significant disparity in perceptions of resident preoperative preparation, and intraoperative and postoperative feedback between residents and surgical faculty [13]. Much of this disparity was centered on the residents being more focused on the technical aspects of the procedure while faculty felt that natural history of disease and patient outcomes were more important [14]. Similarly, Juve et al. found that faculty members reported spending significant time teaching on patient issues related to the cases that they were actively managing with residents, but residents felt that this interaction was part of patient care responsibilities rather than teaching and defined teaching as a discussion of topics beyond those associated with patients that they were managing [15]. They found that use of teaching tool for residents and faculty members to meet the residents’ desire beyond the scope of active patient care was associated with improvement and better alignment of resident and faculty perceptions [15].

### Limitations

These findings may not be generalizable for other rotations or institutional settings. Billing data, used for objective confirmation of resident evaluations, may be incomplete. Although we instructed residents to evaluate the educational value of the service, we cannot separate less work hours and popularity from ratings of educational value of the rotation.

Resident perceptions fall under the category of stakeholder satisfaction or perception of educational value. However, resident perception is important, as it is a marker of what is important to the resident and what knowledge is retained and can be applied [16]. Importantly, resident perceptions and faculty member perceptions on teaching and feedback and objectives are often not aligned making formal acknowledgment of resident perceptions an important part of graduate medical education.

Additional limitations include different numbers of PGY1, PGY2, and PGY3 residents during FY2013, 2014, and 2015. By calculating the average number of patients seen per resident as a conglomerate rather than by individual resident, the individual differences are reduced. While we used a historical control, no appropriate concurrent control exists. The year prior to implementation with the same residency and same curriculum served as our baseline group. Due to the nature of the evaluation, there is risk of sampling bias due to annual variability of the make-up of the resident class. In addition, attending physicians were not surveyed during these time periods to see if their perceptions of resident learning paralleled the subjective resident experience.

## Conclusions

Assigning additional personnel to off-load the resident clinics led to improvements in resident perceptions of educational value of a medicine consultation rotation. Use of both staff physicians and APP were associated with clear reductions in resident workload in contrast to other efforts aimed at reducing work hours. Attention to resident workload may help improve resident satisfaction and resident-based faculty evaluations. Adding an APP may improve resident experiences on rotations with overwhelming clinical workloads.

## Additional files

Additional file 1: Billing codes. Current Procedural Terminology (CPT) code for both staff only and resident and staff billing codes for initial consults and follow up consults. (DOCX 13 kb)
Additional file 2: Surgical comanagement (SCM) resident evaluation survey form. The paper copy of the electronic survey addressed to residents to evaluate the surgical comangement rotation. (DOCX 15 kb)

## Abbreviations

ACGME: Accreditation council for graduate medical education
APP: Advanced practice provider
FY: Fiscal year
SCM: Surgical comanagement
UIHC: University of Iowa Hospitals and Clinics
