# Supplementary material for: Impact of adding additional providers to resident workload and the resident experience on a medical consultation rotation
Source: BMC Med Educ. 2017 Feb 22;17:44. doi: 10.1186/s12909-017-0874-7 (PMC5322644; doi:10.1186/s12909-017-0874-7)
Supplement: Additional file 1: — Billing codes. Current Procedural Terminology (CPT) code for both staff only and resident and staff billing codes for initial consults and follow up consults. (DOCX 13 kb) [file 12909_2017_874_MOESM1_ESM.docx]

Billing codes: Outpatient level 1 99241gc, Outpatient level 2 99242gc, outpatient level 3 99243gc, outpatient level 4 99244gc, outpatient level 5 99245gc, inpatient initial consult low 99253gc, inpatient initial consult moderate 99254gc, inpatient initial consult high 99255gc, daily care, low 99231gc, daily care moderate 99232gc, daily code high 99233gc

Staff only codes Outpatient level 1 99241, Outpatient level 2 99242, outpatient level 3 99243gc, outpatient level 4 99244gc, outpatient level 5 99245, inpatient initial consult low 99253, inpatient initial consult moderate 99254, inpatient initial consult high 99255, daily care, low 99231, daily care moderate 99232, daily code high 99233
