# Supplementary material for: Impact of adding additional providers to resident workload and the resident experience on a medical consultation rotation
Source: BMC Med Educ. 2017 Feb 22;17:44. doi: 10.1186/s12909-017-0874-7 (PMC5322644; doi:10.1186/s12909-017-0874-7)
Supplement: Additional file 2: — Surgical comanagement (SCM) resident evaluation survey form. The paper copy of the electronic survey addressed to residents to evaluate the surgical comangement rotation. (DOCX 15 kb) [file 12909_2017_874_MOESM2_ESM.docx]

Evaluator: ________________________

Service: Cons SCM

Rotation (dates): _________________________

Issue date:_________________________

**Insufficient contact to evaluate (delete evaluation)

**Clinical experience**

1. Adequacy of patient volume *

| Too many | Just right | Too few |
| --- | --- | --- |
| o | o | o |

1. Opportunity to perform procedures with needed supervision*

| Too many | Just right | Too few |
| --- | --- | --- |
| o | o | o |

1. Appropriateness balance between responsibility and supervision*

| Always or usually | Sometimes | Rarely or never |
| --- | --- | --- |
| o | o | o |

1. Appropriateness of patient case mix *

| Always or usually | Sometimes | Rarely or never |
| --- | --- | --- |
| o | o | o |

Educational experience

1. The rotation specific curricular goals were met*

| Strongly Disagree | Disagree | Uncertain | Agree | Strongly agree |
| --- | --- | --- | --- | --- |
| o | o | o | o | o |

1. Overall educational value of this clinical activity: *

| Poor | Fair | Good | Very good | Excellent |
| --- | --- | --- | --- | --- |
| o | o | o | o | o |

Rotation Strengths (free text)

|  |
| --- |

Rotation Weaknesses (free text)

|  |
| --- |

Comments (free text)

|  |
| --- |

*required field
